# Supplementation With Whey Peptide Rich in β-Lactolin Improves Cognitive Performance in Healthy Older Adults: A Randomized, Double-Blind, Placebo-Controlled Study

**DOI:** 10.3389/fnins.2019.00399

**Published:** 2019-04-24

**Authors:** Masahiro Kita, Keiko Kobayashi, Kuniaki Obara, Takashi Koikeda, Satoshi Umeda, Yasuhisa Ano

**Affiliations:** ^1^Research Laboratories for Health Science and Food Technologies, Kirin Company, Ltd., Yokohama, Japan; ^2^Shiba Palace Clinic, Tokyo, Japan; ^3^Department of Psychology, Keio University, Tokyo, Japan

**Keywords:** dairy food, whey peptide, memory, attention, age-related cognitive decline

## Abstract

Epidemiological reports showed that consumptions of fermented dairy products are beneficial for cognitive decline in elderly. Our previous preclinical studies have demonstrated that intakes of whey peptide rich in the β-lactolin [β-lactopeptide of glycine-thereonine-tryptophan-tyrosine (GTWY)] improve memory and attention by regulating monoamine system, and clinical study using neuropsychological test suggested that consumptions with GTWY-rich whey peptide enhance cognitive performance associated with the frontal cortex activity. However, corresponding interventional studies in humans are limited. Objectives: to evaluate the effects of the whey peptide on cognitive functions in healthy older adults using a randomized, double-blinded, placebo-controlled trial design. 114 healthy subjects aged 50–75 were supplemented with the whey peptide or placebo for 12 weeks, and changes in cognitive function were assessed using neuropsychological tests at weeks 0, 6, and 12 of the intervention. Neuropsychological tests included assessments for memory functions (subtests from Wechsler memory scale-revised, standard verbal paired-associate learning test, and recognition memory test for faces), assessments for attention (cancelation and detection tests), and assessments for general cognitive functions (repeatable battery for assessments of neuropsychological status). Cerebral blood flow was also assessed using near-infrared spectroscopy (NIRS) after 6 weeks of intervention. This study was registered on the 19 November, 2017 in the database of the University Hospital Medical Information Network (UMIN) prior to enrollment of subjects (Registration No. UMIN000030461: https://www.umin.ac.jp/ctr/index-j.htm). In the whey peptide group, visual paired-associates I and visual cancelation tests were significantly improved compared with those in the placebo group at weeks 6 and 12 of the intervention, respectively. Visuospatial and constructional scores of the repeatable battery for assessments of neuropsychological status and standard verbal paired-associate learning tests (S-PA) also tended to be improved by the intervention at week 12. Daily intakes of GTWY-rich whey peptide show beneficial effects on cognitive performance, especially associative learning memory and control of attention, in healthy older adults and might prevent age-related cognitive declines.

## Introduction

People with age-related cognitive declines or dementia are rapidly increasing in number globally, due to increasingly aged populations. It is estimated that the number of patients with dementia will reach 130 million by 2050 (Prince et al., 2015). Due to lacking therapeutic approaches for dementia, including Alzheimer’s disease, preventative approaches in daily life, e.g., dietary control, have been considered widely.

Previous epidemiological studies show that daily consumption of dairy products prevents age-related cognitive declines (Crichton et al., 2010; Ozawa et al., 2013; Ogata et al., 2016). Some dairy products have also been shown to prevent cognitive declines in interventional studies in rodents and humans. Our group previously demonstrated that camembert cheese, a dairy product fermented with fungi, prevents the development of Alzheimer’s pathology using the transgenic model mice (Ano et al., 2015). Other groups showed that consumption of whey proteins (more than 20 g per day), which are abundant in supernatants of yogurt and are byproducts of cheese, improve memory in elderly and stress-vulnerable subjects (Markus et al., 2000; Kaplan et al., 2001). These studies collectively suggest that some components in dairy products ameliorate cognitive declines and prevent dementia.

Recently, we identified tryptophan-tyrosine (WY)-related peptides, including the β-lactopeptide of glycine-threonine-tryptophan-tyrosine (GTWY), β-lactolin, derived from β-lactoglobulin in whey proteins digested by specific enzymes. WY-related peptides such as GTWY are abundant in fermented dairy products, including camembert cheese. The GTWY peptide and whey peptides with high proportions of GTWY have been shown to increase monoamine levels in the frontal cortex and hippocampus and improve spatial working memory and attention in aged mice and pharmacologically-induced amnesia mice (Ano et al., 2018). We also showed that supplementation with 1 g of GTWY-rich whey peptide improved the score of verbal fluency test and Stroop test evaluating memory, attention, and executive functions in healthy middle-aged adults (45–64 year-old) with high subjective fatigue compared with placebo control (Kita et al., 2018). These studies suggested that consumption of the GTWY-rich whey peptide is associated with the activation of frontal cortex, especially dorsolateral prefrontal cortex (DLPFC) regulating memory retrieval and executive function. On the other hand, the evidence of these effects on memory and executive function remains limited to relatively middle-age adults with high subjective fatigue, and the underling mechanisms have not been elucidated in humans.

We conducted this study in order to validate the conclusions of the previous clinical trial and to extend the evidence of the whey peptide as far as older adults. In this study, we examined the effects of GTWY-rich whey peptide on cognitive functions in healthy older adults (50–75-year-old) in a randomized, placebo-controlled, double-blind, parallel-group comparative study design. We mainly used some of the Wechsler memory scale-revised (WMS-R) (Koike and Sugishita, 2014) and the standard verbal paired-associate learning test (S-PA) (JSfHB, 2011) to evaluate memory functions, and the clinical assessment for attention (CAT) (Kato, 2006) to evaluate the ability of attention control and executive function. In addition, we measured cerebral blood flow and associated these measurements with neuropsychological tests.

## Materials and Methods

### Subjects

We recruited 294 Japanese-speaking healthy older adults aged 50–75 years, with self-awareness of decline in brain functions such as forgetfulness (e.g., often forgetting the name of people and object) and carelessness. Subjects with relatively low scores in repeatable battery tests of neuropsychological status (RBANS) were preferentially included. Subjects were excluded if they had (Prince et al., 2015) visual or hearing impediments (Crichton et al., 2010) suspected dementia (Ozawa et al., 2013) anamnesis of cranial nerve disease (Ogata et al., 2016) diagnosis of depression or depressive symptoms (Ano et al., 2015) menopausal symptoms or current hormone treatments (Kaplan et al., 2001) irregular lifestyle such as shift work (Markus et al., 2000) habits of high alcohol consumption (>20 g/day) (Ano et al., 2018) cigarette smoking habits (Kita et al., 2018) experience of neuropsychological tests within the past year (Koike and Sugishita, 2014) current treatments for cognitive function (JSfHB, 2011) regular consumption of drugs or health foods that may affect cognitive function (>once a week) (Kato, 2006) regular consumption of protein supplements (>once a week) (Nakamori et al., 2006) anamnesis of severe diseases requiring regular treatments (Ohsawa et al., 2006) allergy or sensitivity to milk (Yamashima et al., 2002) pregnancy or were breastfeeding. Inclusion and exclusion criteria were checked during screening steps using a questionnaire and in mini-mental state examinations (MMSEs), subject diaries, clinical examinations, and interviews by the principal investigator.

Previous studies of other functional foods required 20 to 30 subjects to detect significant differences in RBANS (α = 0.05) (Nakamori et al., 2006; Ohsawa et al., 2006). To ensure sufficient statistical power after stratification, at least 50 subjects were required in each group.

### Experimental Supplements

The test tablets containing 1 g of whey peptide, which included 1.6 mg of β-lactopeptide of GTWY, β-lactolin, were prepared by Kirin, Co., Ltd. Tablets were ingested by the test group every day for 12 weeks. Whey peptide was substituted with the same amount of maltodextrin in placebo tablets. The test and placebo tablets were indistinguishable by size, shape, or taste.

### Procedures

This study was performed according to a randomized, placebo-controlled, double-blind, parallel-group comparative design. Questionnaires for inclusion and exclusion criteria and lifestyle data, subject dairies, measurements of blood pressure, height, and body weight, MMSE, and RBANS were performed during the first screening step, and clinical examinations and medical interview for assessments of safety by principal investigator were performed during the second screening step. RBANS is the standardized neuropsychological test, and suitable for easy assessment of general cognitive domains. Selected subjects were randomly allocated to the whey peptide or placebo groups by a computer program that ensured similar age, sex, and RBANS scores between study groups. The study group allocator was not involved in assessments of eligibility, data collection, or analysis. Subjects, research staff, and outcome assessors were blinded to group allocations until data analyses had been completed. Neuropsychological tests were performed after 0, 6, and 12 weeks of intervention. At week 6 of the intervention period, cerebral blood flow was measured using near-infrared spectroscopy (NIRS) before and after neuropsychological tests, which will be described in detail in a subsequent publication. Clinical examinations of safety were performed after 12 weeks of intervention. Subjects were instructed to maintain regular lifestyles and avoid taking any drugs, health foods, or protein supplements that may affect cognitive performance during the study. Compliance was monitored using subject diaries. On the day of neuropsychological tests, subjects were instructed to completely avoid consuming any foods or beverages containing caffeine, and to avoid ingesting any foods and beverages, except for water, for 4 h prior to the start of neuropsychological tests. Data were collected at Shiba palace clinic (Tokyo, Japan) between January 2018 and June 2018.

### Neuropsychological Tests (Primary Outcomes)

To evaluate multiple cognitive domains, we performed the Japanese version of RBANS (Yamashima et al., 2002), some of the Japanese version of the Wechsler memory scale-revised (WMS-R) (Koike and Sugishita, 2014), the clinical assessment for attention (CAT) (Kato, 2006), the standard verbal paired-associate learning test (S-PA) (JSfHB, 2011), and the recognition memory test for faces (RMT-F).

Repeatable battery for assessments of neuropsychological status comprises 12 subtests and evaluates the following five indexes of cognitive domains: immediate memory, visuospatial/constructional, language, attention, and delayed memory. Age-based index scores were calculated for each index.

We also conducted design memory, logical memory I and II, spatial span, and visual paired-associate I and II tests from the WMS-R. Design memory assesses visual memory and requires subjects to memorize abstract designs and immediately recognize them in multiple obstructing designs. Logical memory assessments of short-term verbal memory required subjects to recall orally-presented short stories immediately (Logical memory I) and 30 min later (Logical memory II). In spatial span assessments of spatial attention, the assessor indicates a sequence of printed boxes (on paper) by touching them, and subjects are then required to touch the boxes in the same (forward) and reversed (backward) orders. Total scores for forward and backward accuracy are then calculated. In visual paired-associate assessments of visual associative learning, subjects were presented pictures with associated colors, and are then required to point to the correct colors when presented each of the pictures, immediately (Visual paired-associate I) and 30 min later (Visual paired-associate II).

**FIGURE 1:**
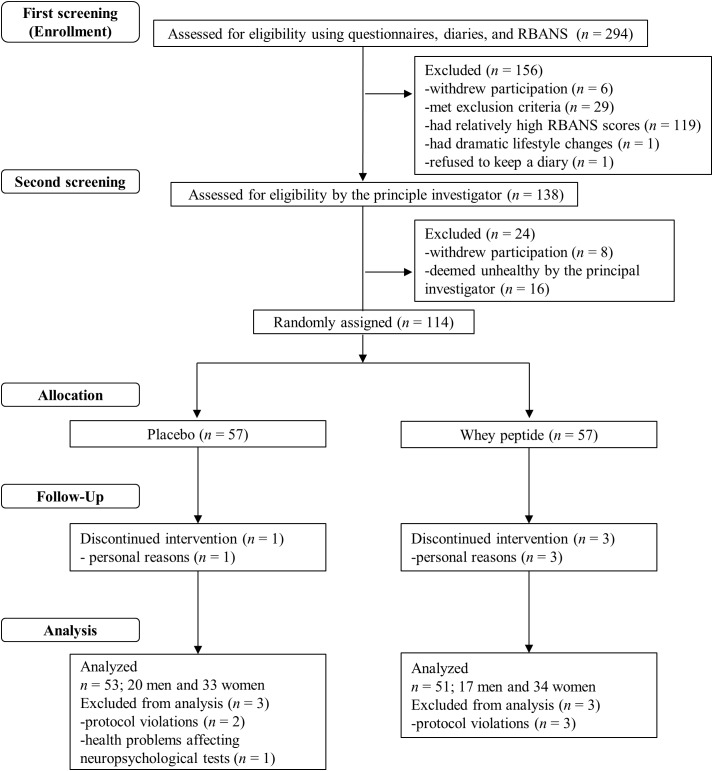
Flow chart of the subject selection process. Out of 294 subjects who were screened, 114 subjects were included in the study. These subjects were randomly allocated to whey peptide (*n* = 57) and placebo (*n* = 57) groups. Three subjects in the whey peptide group and one subject in the placebo group dropped out of the study during the intervention. Three subjects in the whey peptide group and three in the placebo group were excluded from analysis, leaving 51 and 53 subjects in whey peptide and placebo groups, respectively, for analysis.

**FIGURE 2:**
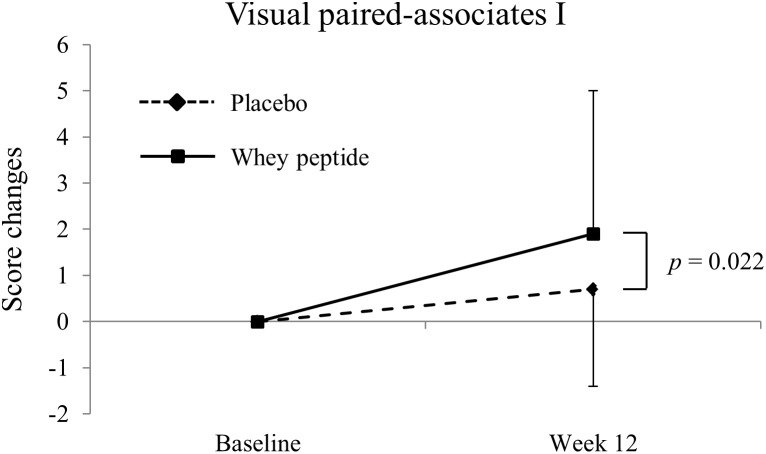
Changes of visual paired-associates I from baseline. The solid line shows the whey peptide group (*n* = 51), and the dashed line shows the placebo group (*n* = 53). The data are presented as the mean with SD. The *p*-value shows the results of between-group comparison, performed by unpaired *t*-test. *p* < 0.05 was considered as significant difference.

Visual cancelation tests and auditory detection test from CAT were performed to assess visual or auditory selective attention, respectively. Visual cancelation tests required subjects to delete targets (Figure 1, 2, number of three, Japanese “ka”) as early as possible in interference stimulus. In auditory detection tests, Japanese letters were orally and randomly presented, and subjects were required to respond to the target letter (Japanese “to”).

Verbal associative learning was assessed using S-PA, in which subjects were presented pairs of words that were semantically related or unrelated. Subsequently, subjects were presented with one of each pair of words and were required to correctly recall the other.

Cancelation and detection tests and S-PA were repeated at 6 and 12 weeks after the start of the intervention. To avoid learning effects, other neuropsychological tests were repeated at only the 12 week time point. RBANS and other neuropsychological tests were performed on separate days during week 12 of the study.

Recognition memory test for faces was performed to assess the memory for faces. Subjects were presented 32 unfamiliar human faces from the database of The Karolinska Directed Emotional Faces in the encoding phase (Lundqvist et al., 1998). In the recognition phase, subjects were shown the presented 32 faces and new 32 distractor faces, and made old/new response (Warrington, 1984). Corrected recognition score (hit rate – false alarm rate) were collected.

### Safety Assessment

Safety assessments were made with consideration of subjective symptoms, findings from interviews by the principal investigator, and measurements of body weight, body mass index (BMI), blood pressure, and pulse. Blood and urine samples were also collected.

### Statistical Analysis

Data are presented as means ± standard deviations (SD). Statistical analyses were performed using Dr.SPSS II for Windows (IBM, Somers, NY, United States) and differences between groups were identified using unpaired *t*-tests. Baseline data from cancelation and detection tests and S-PA were compared with those from after the intervention using Dunnett’s test, and comparisons of other neuropsychological tests were examined with paired *t*-test. Differences were considered significant when *p* < 0.05.

### Ethics and Registration

This study was carried out in accordance with the recommendations of Ethical Guidelines for Medical and Health Research Involving Human Subjects, Ministry of Health, Labor and Welfare’ with written informed consent from all subjects. All subjects gave written informed consent in accordance with the Declaration of Helsinki. The protocol was approved by the ethics committee of Shiba palace clinic. The study was registered on the 19 November, 2017 in the database of the University Hospital Medical Information Network (UMIN) prior to enrollment of subjects (Registration No. UMIN000030461; Registration title. A study for the effect of intake of test foods on cognitive functions).

## Results

### Baseline Characteristics of the Study Group

A flow chart of the subject selection process is presented in Figure 1. Following the first screening step, 138 subjects were included and 156 subjects were excluded due to withdrawal of participation (*n* = 6), suspected dementia (MMSE < 24; *n* = 5), regular consumption of health foods that may affect cognitive function (*n* = 12), high habitual alcohol consumption (*n* = 3), irregular lifestyle (*n* = 8), experience of neuropsychological tests within 1 year (*n* = 1), dramatic lifestyle change during the screening step (*n* = 1), refusal to keep the subject diary (*n* = 1), and relatively high RBANS scores (*n* = 119). During the second screening step, 24 subjects were excluded due to withdrawal of participation (*n* = 8) or classification as unhealthy by the principal investigator (*n* = 16). The remaining 114 subjects were randomly allocated to whey peptide and placebo groups and were supplemented for 12 weeks. Three subjects in the whey peptide group and one subject in the placebo group withdrew from the study due to personal reasons, leaving 54 and 56 subjects in whey peptide and placebo groups, respectively. After completion of all tests, three subjects in the whey peptide group were excluded from analysis due to protocol violations (*n* = 3), and three in the placebo group were excluded due to protocol violations (*n* = 2) or health problems that could affect neuropsychological tests (*n* = 1). Finally, 51 subjects in the whey peptide group and 53 subjects in the placebo group were analyzed, as shown in Table 1.

**Table 1:** Subject characteristics at baseline (week 0).

Characteristics	Placebo	Whey peptide	*p*
Age	61.2 ± 5.6	60.7 ± 5.7	0.703
Male/female	20/33	17/34	0.639
Body mass index (kg/m^2^)	22.1 ± 3.3	21.6 ± 2.8	0.414
Education years	9.38 ± 2.21	9.51 ± 2.98	0.797
MMSE score	28.7 ± 1.4	28.5 ± 1.5	0.565
RBANS total scale	54.5 ± 12.1	53.7 ± 14.1	0.772

*Data are presented as means ± standard deviations (SD) for placebo (n = 53) and whey peptide (n = 51) groups; p-values were calculated using unpaired t-tests, except those for male/female ratios was calculated using χ^2^ tests.*

### Primary Outcome

#### Neuropsychological Tests From WMS-R and S-PA

Changes in WMS-R and S-PA neuropsychological tests from baseline (week 0) to week 12 are shown in Tables 2, 3, respectively. Post-intervention WMS-R neuropsychological tests showed significantly increased scores for logical memory I and II and visual paired-associates I in whey peptide and placebo groups compared with those at baseline, and these changes were higher in the whey peptide group than in the placebo group. In particular, changes in visual paired-associates I from baseline were significantly higher in the whey peptide group than in the placebo group (*p* = 0.022; Figure 2 and Table 2). In S-PA tests, score changes were significantly increased for unrelated pairs (2^nd^) after the 12-week intervention in the whey peptide group, compared with those at baseline, but no increases in these scores were observed in the placebo group. Changes in S-PA scores for unrelated pairs (2^nd^) tended to be higher in the whey peptide group than in the placebo group at week 12 of the intervention (*p* = 0.051; Table 3). Changes in the other scores for unrelated pairs (1^st^ and 3^rd^) were also higher in whey peptide group than in the placebo group (Table 3). Taken together, these data indicate that the consumption of whey peptide might especially improve the recall of paired-associative learning.

**Table 2:** Changes in Wechsler memory scale-revised (WMS-R) tests.

	Group	Week 12	*p*
Design memory	Placebo Whey peptide	0.28 ± 1.38 0.24 ± 1.73	0.876
Logical memory I	Placebo Whey peptide	1.5 ± 4.8^∗^ 2.3 ± 5.1^∗∗^	0.366
Visual paired-associates I	Placebo Whey peptide	0.7 ± 2.1^∗^ 1.9 ± 3.1^∗∗^	0.022
Spatial span	Placebo Whey peptide	0.5 ± 2.7 −0.2 ± 2.4	0.193
Logical memory II	Placebo Whey peptide	2.5 ± 5.2^∗∗^ 3.0 ± 5.3^∗∗^	0.649
Visual paired-associates II	Placebo Whey peptide	0.02 ± 0.80 0.10 ± 1.00	0.656

*Data are presented as means ± SD for placebo (n = 53) and the whey peptide (n = 51) groups. Group differences were identified using unpaired t-tests. Significant differences between baseline and week 12 were identified using paired t-tests; ^∗^p < 0.05 and ^∗∗^p < 0.01.*

**Table 3:** Changes in standard verbal paired-associate learning (S-PA) and clinical assessment for attention (CAT) tests.

	Group	Week 6	*p*	Week 12	*p*
**S-PA**					
Unrelated pairs (1^st^)	Placebo Whey peptide	−0.15 ± 2.23 −0.39 ± 2.65	0.616	0.30 ± 2.23 0.57 ± 2.43	0.561
Unrelated pairs (2^nd^)	Placebo Whey peptide	−1.25 ± 2.43^∗∗^ −0.49 ± 2.73	0.139	0.11 ± 1.94 1.08 ± 2.93^∗∗^	0.051
Unrelated pairs (3^rd^)	Placebo Whey peptide	−0.96 ± 2.46^∗∗^ −0.55 ± 2.34	0.382	0.17 ± 2.35 0.55 ± 2.63	0.440
**Visual cancelation task**					
Figure 1 – required time (s)	Placebo Whey peptide	−2.7 ± 4.3^∗∗^ −4.9 ± 8.7^∗∗^	0.106	−3.5 ± 4.4^∗∗^ −5.2 ± 8.1^∗∗^	0.192
Figure 1 – Accuracy (%)	Placebo Whey peptide	0.46 ± 2.60 0.86 ± 2.39^∗^	0.420	0.56 ± 3.22 1.14 ± 2.76^∗∗^	0.334
Figure 1 – Hitting ratio (%)	Placebo Whey peptide	−0.03 ± 0.42 −0.03 ± 0.24	0.992	0.03 ± 0.24 −0.03 ± 0.24	0.160
Figure 2 – required time (s)	Placebo Whey peptide	−2.1 ± 5.9^∗^ −5.9 ± 10.6^∗∗^	0.024	−3.6 ± 5.7^∗∗^ −6.8 ± 11.0^∗∗^	0.066
Figure 2 – Accuracy (%)	Placebo Whey peptide	−0.40 ± 3.60 0.62 ± 4.19	0.187	0.27 ± 2.24 0.86 ± 4.19	0.366
Figure 2 – Hitting ratio (%)	Placebo Whey peptide	0.04 ± 0.96 0.04 ± 0.91	0.998	0.11 ± 0.67 0.07 ± 0.88	0.834
Number “3” – required time (s)	Placebo Whey peptide	−3.0 ± 6.7^∗∗^ −6.3 ± 14.3^∗∗^	0.148	−2.8 ± 7.3^∗∗^ −5.5 ± 14.6^∗∗^	0.237
Number “3” – Accuracy (%)	Placebo Whey peptide	0.45 ± 2.26 0.12 ± 1.67	0.406	0.55 ± 2.69 0.41 ± 1.88	0.771
Number “3” – Hitting ratio (%)	Placebo Whey peptide	−0.00 ± 0.25 −0.03 ± 0.17	0.416	−0.02 ± 0.27 −0.02 ± 0.13	0.970
Japanese “ka” – required time (s)	Placebo Whey peptide	−3.6 ± 8.6^∗∗^ −7.2 ± 14.7^∗∗^	0.127	−3.3 ± 8.9^∗∗^ −6.1 ± 15.4^∗∗^	0.250
Japanese “ka” – Accuracy (%)	Placebo Whey peptide	0.65 ± 3.15 0.93 ± 2.90	0.634	1.01 ± 2.86^∗^ 1.17 ± 4.43^∗^	0.827
Japanese “ka” – Hitting ratio (%)	Placebo Whey peptide	−0.05 ± 0.21 0.04 ± 0.31	0.091	−0.07 ± 0.24 0.04 ± 0.44	0.130
**Auditory detection task**					
Accuracy (%)	Placebo Whey peptide	−1.5 ± 8.5 0.5 ± 4.8	0.149	−0.30 ± 6.6 −0.55 ± 5.7	0.838
Hitting ratio (%)	Placebo Whey peptide	4.1 ± 16.2 5.2 ± 21.6	0.763	7.9 ± 18.6^∗∗^ 6.9 ± 16.9^∗^	0.775

*Data are presented as means ± SD for placebo (n = 53) and whey peptide (n = 51) groups. Group differences were identified using unpaired t-tests. Changes from baseline were identified using Dunnet’s tests; ^∗^p < 0.05 and ^∗∗^p < 0.01.*

#### Neuropsychological Tests From CAT

**FIGURE 3:**
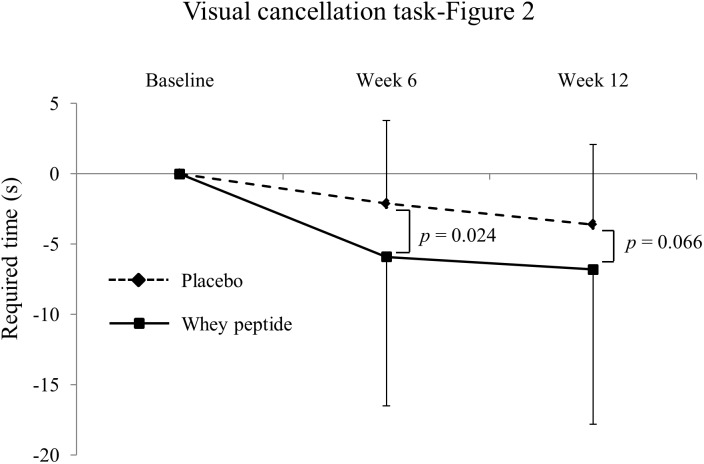
Changes of required time in visual cancelation task-Figure 2 from baseline. The solid line shows the whey peptide group (*n* = 51), and the dashed line shows the placebo group (*n* = 53). The data are presented as the mean with SD. The *p*-value shows the results of between-group comparison, performed by unpaired *t*-test. *p* < 0.05 was considered as significant difference.

Changes in neuropsychological test results from CAT are shown in Table 3. In visual cancelation tests, required times were significantly decreased by interventions in both groups compared with those at baseline, and changes from baseline were greater in the whey peptide group than in the placebo group. Moreover, changes in required times (Figure 2 subtest) in the whey peptide group were significantly greater than those in the placebo group at week 6 of the intervention and tended to be greater at week 12 of interventions (*p* = 0.024 and *p* = 0.066, respectively; Figure 3 and Table 3). Accuracy in the subtest in Figure 1 was significantly increased at weeks 6 and 12 compared with that at baseline in the whey peptide group, but not in the placebo group. Furthermore, changes in hitting ratios for the Japanese “ka” subtest tended to be higher at week 6 in the whey peptide group than in the placebo group (*p* = 0.091; Table 3). In contrast, no significant differences in accuracy and hitting ratios of auditory detection tasks were identified between intervention groups. These results indicate that consumption of whey peptide might improve the performance of attention for visual information with increasing the accuracy.

#### Repeatable Battery for Assessments of Neuropsychological Status

As shown in Supplementary Table 1, RBANS for visuospatial/constructional indexes tended to be higher in the whey peptide group than in the placebo group at week 12 of the intervention (*p* = 0.099). Scores from the figure copy test, which is one of the subtests of visiospatial/constructional assessments, were significantly higher in the whey peptide group than in the placebo group at week 12. Yet no significant differences in other RBANS subtests were identified between groups. These results indicate that consumption of whey peptide improves spatial recognition.

#### Safety Assessments and Compliance

To evaluate the safety of whey peptide, subjective dairies were maintained, and clinical examinations and medical interviews were performed by the principal investigator. Thirty subjects in the placebo group and 30 subjects in the whey peptide group reported adverse events during the study, but none of these were related to the interventions. Some clinical values changed slightly from baseline, but these were deemed clinically insignificant. Compliance with interventions was very high, with average tablet consumption rates of 99.9% in the placebo group and 99.8% in the whey peptide group.

## Discussion

In this randomized, double-blind, placebo-controlled trial, we evaluated the effects of daily supplements with whey peptide containing 1.6 mg of GTWY, β-lactolin, on cognitive functions in healthy older adults. Subjects in the whey peptide group showed greater improvements in associative learning and selective attention compared with those in the placebo group, suggesting that whey peptide consumption might improve some aspects of memory and control of attention in healthy older adults.

In the present study, we employed some parts of the WMS-R, S-PA, and RMT-F to evaluate memory function and some parts of the CAT to assess attention. Compared to the placebo group, scores from paired-associates tests (both visual paired associates of WMS-R and S-PA) were increased in the whey peptide group more than in the other types of memory tests, suggesting that whey peptide improves memory for associative learning. In the retrieval process of paired-associate tests, subjects received cues to recall prior associations. It is generally accepted that cued recall requires less neural activity than free-recall, for which subjects recall without cues (Rainer et al., 1999). In addition, immediate recall was more enhanced by whey peptide consumption than delayed recall, suggesting that whey peptide is associated with memory recall requiring attention.

In measurements of several attention functions, whey peptide especially improved the time required for visual cancelation tests in CAT without changing accuracy, suggesting that whey peptide might improve the control of selective attention. Selective attention requires focus on important stimuli in focus-obstructing environments. This capacity is necessary for humans to ignore unimportant information in favor of important considerations. The present changes in selective attention differed significantly between intervention groups after 6 weeks and tended to be significant at week 12. Because the same version of the visual cancelation test was used at baseline and at weeks 6 and 12, learning effects may have weakened differences between placebo and whey peptide groups at week 12. Scores of paired-associate (visual and verbal) and visual cancelation tests have been shown to decline with aging in multiple studies (Uttl and Pilkenton-Taylor, 2001; JSfHB, 2011; Koike and Sugishita, 2014), and are widely used to discriminate between neurodegenerative disorders such as Alzheimer’s disease (Amieva et al., 2004; JSfHB, 2011). We observed a weak negative correlation between age and baseline scores of visual paired-associates I (*r* = −0.27), suggesting that the daily intake of whey peptide ameliorates age-related impairments of attention.

Memory functions are generally performed in the hippocampus and frontal cortex, but the frontal cortex, especially the dorsolateral prefrontal cortex (DLPFC), plays major roles in associative learning. Functional magnetic resonance imaging (MRI) studies revealed that DLPFC activities increase during associative learning processes, but do not increase during item-specific learning processes (Blumenfeld et al., 2011; Hawco et al., 2013). Hence, the DLPFC is critical for building relationships between items, and this process may facilitate memory retrieval (Blumenfeld et al., 2011; Hawco et al., 2013). As with associative learning, selective attention is dependent on the prefrontal cortex and requires large subjective efforts (Toichi et al., 2004). Accordingly, functional MRI studies showed important roles of the DLPFC and the anterior cingulate cortex in these processes (Prado et al., 2011). Our results are consistent with a previous clinical trial showing that GTWY-rich whey peptide improves verbal fluency and moderates the inhibition of executive functions that largely depend on the prefrontal cortex (Kita et al., 2018). Taken together, these data suggest that GTWY-rich whey peptide is associated with the activity of the prefrontal cortex, especially in the DLPFC. In addition, the dopamine D1 receptor (D1R) in the prefrontal cortex has been associated with both associative learning and selective attention (Noudoost and Moore, 2011; Puig and Miller, 2012), and we previously showed that the effects of GTWY are mediated by D1R in mice (Ano et al., 2018). GTWY were quickly absorbed and partially delivered to the brain (Ano et al., 2018). GTWY inhibited the activity of dopamine degrading enzyme, monoamine oxidase B, and increased the level of cerebral dopamine (Ano et al., 2018). The memory and attention-enhancing activity of GTWY was attenuated by the treatment of D1R inhibitor (Ano et al., 2018). Thus, the effects of GTWY and GTWY-rich whey peptide on the activity of DLPFC via dopaminergic systems may improve cognitive functions, e.g., associative learning and control of attention. Furthermore, the reduction in body weight from the baseline in the whey peptide group at week 6 was significantly larger than in the placebo group (week 6, whey peptide; −0.669 ± 1.301, placebo; −0.175 ± 1.207, *p* = 0.048. week 12, whey peptide; −0.673 ± 1.291, placebo; −0.564 ± 1.419, *p* = 0.685), and the reduction in BMI at week 6 tended to decreased in the whey peptide compared to in the placebo (week 6, whey peptide; −0.253 ± 0.515, placebo; −0.066 ± 0.458, *p* = 0.053; week 12, whey peptide; −0.255 ± 0.513, placebo; −0.204 ± 0.532, *p* = 0.619). It is suggested that body weight and BMI might be associated with cognitive functions (Emiliano et al., 2017). Thus, the changes in body weight might contribute the beneficial effects of the whey peptide on cognitive functions. Further studies are needed to fully elucidate the underling mechanisms.

In agreement with our data, whey peptide reportedly improved cognitive functions in older subjects and in subjects with high psychological fatigue (Kita et al., 2018). In this study, the mean age of subjects was 61.0 years, whereas that in the previous study was 52.0 years. Given the well-described declines in the condition of the prefrontal cortex with aging, Alzheimer’s disease, and cognitive fatigue (Pakkenberg et al., 2003; Lin et al., 2014; Agbangla et al., 2017; Sampath et al., 2017; Wylie et al., 2017), the intake of whey peptide may be more beneficial for the subjects with declines in prefrontal cortex function.

In addition to effects on the prefrontal cortex, consumption of whey peptide may improve the performance of the parietal lobe, as indicated by restoration of spatial recognition in RBANS tests, which require parietal lobe activity (Richard and Sturb, 2005). Consumption of whey peptide, however, did not improve performances in other neuropsychological tests that involve the parietal lobe, such as the design memory test of WMS-R and RMT-F (see Supplementary Table 2). Hence, further research is needed to clarify the effects of whey peptide on parietal lobe function.

This study was limited to a relatively short intervention period, in which progression of age-related cognitive declines may be undetectable. Longer interventions may better elucidate the preventive effects of whey peptide against cognitive declines. We also failed to completely eliminate learning effects in this study. Finally, the mechanisms behind the effects of whey peptide on neuronal activity and connectivity need to be investigated using functional MRI.

In conclusion, the present study shows the association between daily intakes of GTWY-rich whey peptide and improvement of associative learning memory and control of attention in healthy older adults with self-awareness of cognitive declines. This demonstration supports the previous epidemiological, preclinical, and clinical evidence. Daily consumption of whey peptide is easy and safe, so may develop a practical approach to support of age-related cognitive declines.

## Ethics Statement

This study was carried out in accordance with the recommendations of Ethical Guidelines for Medical and Health Research Involving Human Subjects, Ministry of Health, Labor and Welfare’ with written informed consent from all subjects. All subjects gave written informed consent in accordance with the Declaration of Helsinki. The protocol was approved by the ethics committee of Shiba palace clinic.

## Author Contributions

MK, KK, KO, SU, and YA designed the study. TK conducted the study as principal investigator. MK and YA wrote the manuscript. MK assumed primary responsibility for the final content. All authors read and approved the final manuscript.

## Conflict of Interest Statement

MK, KK, KO, and YA were employed by Kirin Company, Limited. The remaining authors declare that the research was conducted in the absence of any commercial or financial relationships that could be construed as a potential conflict of interest.
