# Supplementary material for: Supplementation With Whey Peptide Rich in β-Lactolin Improves Cognitive Performance in Healthy Older Adults: A Randomized, Double-Blind, Placebo-Controlled Study
Source: Front Neurosci. 2019 Apr 24;13:399. doi: 10.3389/fnins.2019.00399 (PMC6491855; doi:10.3389/fnins.2019.00399)
Supplement: Supplementary file 1 [file Table_1.DOCX]

Supplementary Material

**Supplementary table 1. Repeatable battery for assessments of neuropsychological status (RBANS) scores**

|  | **Group** | **Baseline** | ***p*** | **Week 12** | ***p*** |
| --- | --- | --- | --- | --- | --- |
| Total Scale | Placebo  Whey peptide | 54.5 ± 12.1  53.7 ± 14.1 | 0.772 | 59.1 ± 10.3**  59.7 ± 10.2** | 0.734 |
| Immediate Memory | Placebo  Whey peptide | 52.7 ± 12.6  51.8 ± 10.4 | 0.713 | 54.0 ± 10.5  53.3 ± 9.1 | 0.713 |
| Visuospatial Constructional | Placebo  Whey peptide | 51.6 ± 10.6  51.7 ± 9.7 | 0.950 | 50.9 ± 7.8  54.5 ± 13.4 | 0.099 |
| Language | Placebo  Whey peptide | 51.5 ± 7.9  51.6 ± 10.3 | 0.964 | 54.6 ± 9.6*  55.5 ± 8.8** | 0.604 |
| Attention | Placebo  Whey peptide | 55.6 ± 10.2  55.3 ± 13.4 | 0.909 | 60.0 ± 7.6**  61.2 ± 8.7** | 0.462 |
| Delayed Memory | Placebo  Whey peptide | 49.5 ± 14.3  48.7 ± 13.2 | 0.760 | 53.8 ± 9.8*  52.3 ± 8.9* | 0.445 |
| Figure copy | Placebo  Whey peptide | 49.1 ± 9.7  49.9 ± 9.8 | 0.678 | 47.1 ± 11.1  52.3 ± 6.0 | 0.003 |

Data are presented as means ± SD for placebo (*n* = 53) and whey peptide (*n* = 51) groups. Differences between groups were identified using unpaired *t*-tests; **p* < 0.05 and ***p* < 0.01 indicate significant differences between baseline and week 12, as calculated using paired *t*-tests.

**Supplementary table 2. Changes in RMT-F.**

|  | **Group** | **Week 12** | ***P*** |
| --- | --- | --- | --- |
| Corrected recognition score | Placebo  Whey peptide | 0.078 ± 0.138**  0.058 ± 0.119** | 0.426 |

# Data are presented as means ± SD for placebo (*n* = 53) and the whey peptide (*n* = 51) groups. Group differences were identified using unpaired *t*-tests. Significant differences between baseline and week 12 were identified using paired *t*-tests; ***p* < 0.01.
